# Multiple Roles of PLK1 in Mitosis and Meiosis

**DOI:** 10.3390/cells12010187

**Published:** 2023-01-02

**Authors:** Jaroslav Kalous, Daria Aleshkina

**Affiliations:** Institute of Animal Physiology and Genetics, Czech Academy of Sciences, 277 21 Libechov, Czech Republic

**Keywords:** polo-like kinase 1, PLK1, mitosis, meiosis, spindle, oocytes, mRNA translation

## Abstract

Cells are equipped with a diverse network of signaling and regulatory proteins that function as cell cycle regulators and checkpoint proteins to ensure the proper progression of cell division. A key regulator of cell division is polo-like kinase 1 (PLK1), a member of the serine/threonine kinase family that plays an important role in regulating the mitotic and meiotic cell cycle. The phosphorylation of specific substrates mediated by PLK1 controls nuclear envelope breakdown (NEBD), centrosome maturation, proper spindle assembly, chromosome segregation, and cytokinesis. In mammalian oogenesis, PLK1 is essential for resuming meiosis before ovulation and for establishing the meiotic spindle. Among other potential roles, PLK1 regulates the localized translation of spindle-enriched mRNAs by phosphorylating and thereby inhibiting the translational repressor 4E-BP1, a downstream target of the mTOR (mammalian target of rapamycin) pathway. In this review, we summarize the functions of PLK1 in mitosis, meiosis, and cytokinesis and focus on the role of PLK1 in regulating mRNA translation. However, knowledge of the role of PLK1 in the regulation of meiosis remains limited.

## 1. Introduction

Mammalian polo-like kinase 1 (PLK1) belongs to a family of serine/threonine protein kinases that includes five members: PLK1, PLK2, PLK3, PLK4, and PLK5. In mammals, PLK1 plays an important role in mitotic progression [1,2], including key roles in mitotic entry [3,4], centrosome maturation [5,6], chromosome condensation and segregation [7,8,9], coordinating spindle assembly and elongation [3,10], and in cytokinesis [11]. PLK1 expression is related to cell cycle progression; PLK1 expression is low at the G1/S transition, then increases throughout the S phase reaching its maximum in the G2/M phase [12]. Recent data show that PLK1 is important for similar cellular events in meiosis and mitosis (Table 1). Here we focus on describing the expression and activation of PLK1 in mitosis and meiosis and its effects on key steps of cell division. In addition, we also address the direct and indirect roles of PLK1 in translational control.

## 2. Regulation of PLK1 Expression

In somatic cells, PLK1 expression is controlled by the microRNA (miRNA) pathway, as miR-100 directly targets *Plk1* mRNA through interactions with the 3′UTR [36]. PLK1 expression has been shown to be positively regulated by heterogeneous nuclear ribonucleoprotein K (hnRNPK) in various cancer cell lines [37]. hnRNPK competes with microRNA-149-3p (miR-149-3p) and miR-193b-5p which repress PLK1 expression by targeting the 3′UTR of *Plk1* mRNA and therefore hnRNPK upregulates PLK1 expression [37]. Nevertheless, the translational control of *Plk1* mRNA in somatic cells depends on the miRNA pathway, but in mouse oocytes, miRNAs’ role seems redundant for development [38]. Therefore, this suggests that miRNAs do not control *Plk1* mRNA translation during oocyte meiotic maturation.

**Figure 1:**
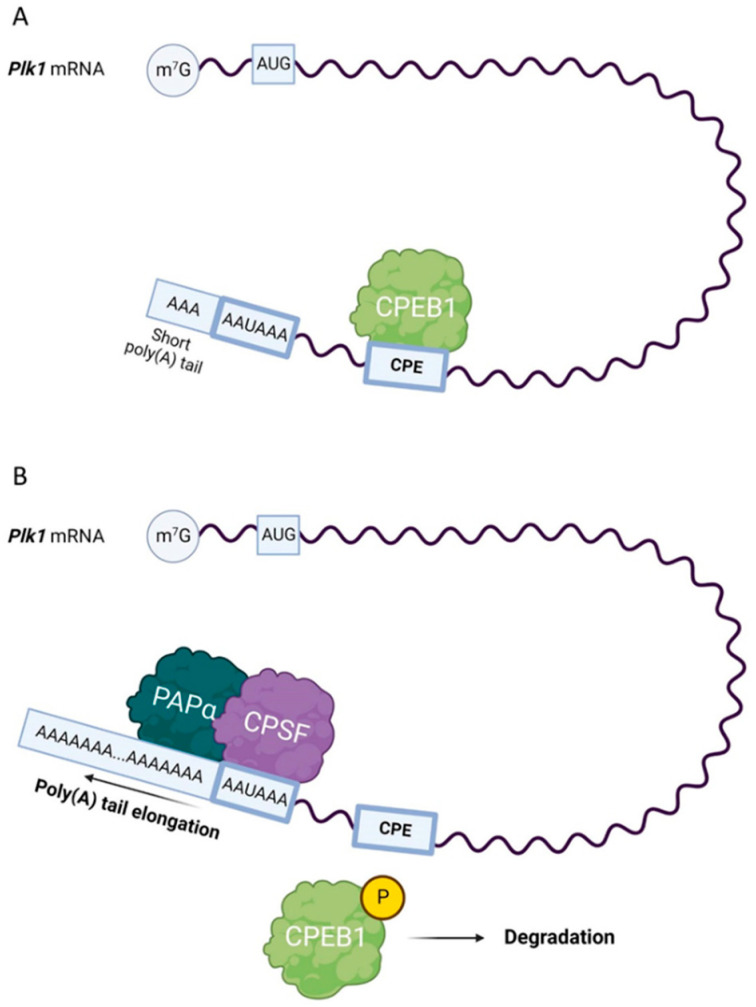
Scheme of *polo-like kinase 1* (*Plk1)* mRNA translation. (**A**) Cytoplasmic polyadenylation element-binding protein 1 (CPEB1) represses the translation of CPE-containing mRNAs with short poly(A) tails, including *Plk1* mRNA. Phosphorylated CPEB1 promotes the binding of the cleavage and polyadenylation specificity factor (CPSF) complex to the hexanucleotide sequence (AAUAAA). (**B**) In mouse oocytes, CPSF recruits poly(A) polymerase α (PAPα), which promotes polyadenylation.

The translation of *Plk1* mRNA in mitosis is also regulated by the interaction between the cytoplasmic polyadenylation element (CPE) present in the 3′ UTRs of mRNAs and CPE-binding protein 1 (CPEB1) and is localized at the mitotic spindle; the translation of *Plk1* mRNA appears to be activated at the G2/M boundary [39] (Figure 1). CPEB1 is activated by Aurora kinase A (AURKA), which is located at mitotic centrosomes [40].

## 3. PLK1 Activation

**Figure 2:**
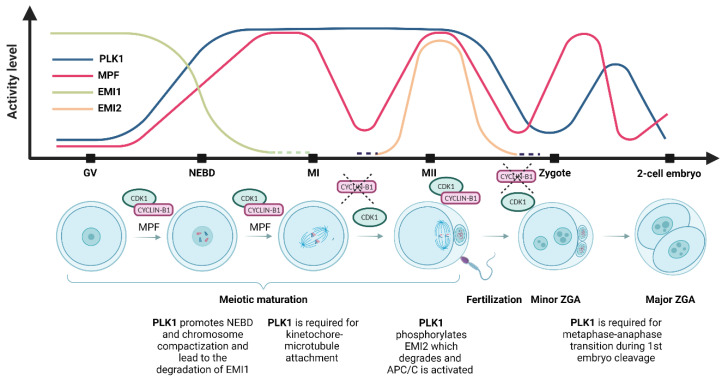
Polo-like kinase 1 (PLK1) and maturation-promoting factor (MPF) activity and the level of early mitotic inhibitor 1 (EMI1) and early mitotic inhibitor 2 (EMI2) during meiotic maturation and oocyte-to-embryo transition. During the first meiotic arrest at the germinal vesicle (GV) stage, PLK1 activity is at very low levels. PLK1 activity starts to increase before nucleus envelope breakdown (NEBD), the inhibition of the anaphase-promoting complex (APC/C) by EMI1 leads to the accumulation of cyclin B, and the activity of the MPF increases. During NEBD, chromosome compaction and nuclear envelope breakdown occur, PLK1 activity rises, MPF activity increases, and EMI1 undergoes destruction immediately after NEBD. At metaphase I (MI), the first metaphase plate is formed, and the first polar body is extruded; PLK1 activity is maintained, MPF reaches maximum activity but is reduced by cyclin B’s destruction by APC/C, and the first polar body is extruded. EMI2 starts to be expressed prior to metaphase II (MII). The second metaphase plate is formed at the MII stage, and meiosis is halted in the second meiotic arrest. PLK1 activity is maintained after the first polar body extrusion, MPF activity is restored to the MI levels, and the oocyte awaits fertilization [49]. In the zygote, after fertilization, EMI2 is degraded and the second polar body is extruded, and both male and female pronuclei are formed. PLK1 activity decreases slowly until the pronucleus stage and is again upregulated after NEBD during the first mitotic division. MPF activity is downregulated by APC/C and restored before the first mitotic division [50]. At the 2-cell embryonic stage, the first mitotic division is completed, PLK1 activity is downregulated, and MPF activity is restored during the subsequent mitotic division.

In vertebrates, PLK1 activity is regulated by the phosphorylation of its threonine residue 210 (Thr210) by AURKA and its cofactor Bora [41,42]. AURKA phosphorylates and activates PLK1 in HeLa cells [42], and the activation of PLK1 by AURKA occurs at mitotic centrosomes [40]. PLK1 activity can be suppressed by inhibitors such as BI2536, which competes with adenosine triphosphate (ATP) for binding to the catalytic domain of PLK1 [43] since the inhibition of PLK1 activity leads to cell cycle arrest in prometaphase. Thus, PLK1 inhibitors are considered potential antimitotic agents for cancer treatment [44,45]. During oocyte meiosis, regulation of PLK1 activity depends on AURKA, as PLK1-Thr 210 phosphorylation was significantly decreased in AURKA knockout oocytes (KO) [46]. In addition, PLK1 activity has been shown to be one of the key substrates of protein kinase A (PKA), and PLK1 activation in mouse oocytes is also regulated by cAMP/PKA signaling [47]. In mouse oocytes, PLK1 activation occurs approximately 30 min before nuclear envelope breakdown (NEBD), and PLK1 activity is maintained through the MI-MII transition until MII arrest. After parthenogenetic activation, the PLK1 phosphorylation and activity decrease, and in the first embryonic mitosis, PLK1 activity increases again, but to lower levels than in meiosis. Subsequently, PLK1 activity decreases to lower levels in the second embryonic interphase than detected during the meiotic decrease of PLK1 activity [14] (Figure 2). Similarly, PLK1 activity is restored during the first mitotic division in embryos from naturally mated mice [48].

## 4. PLK1 Activity and Distribution

In cultured cells, *Plk1* mRNA and protein levels were found to be low throughout the G1 phase, increased during the S phase, and reached maximal levels during the G2 and M phases [19,51]. In HeLa cells, PLK1 activity was low during interphase but reached a maximum during mitosis [52]. The intracellular distribution of PLK1 is cell cycle-dependent. 

PLK1 has a structure that is typical of a PLK family member, i.e., all PLKs are comprised of a highly conserved kinase domain at the amino terminus and a Polo box domain (PBD) at the non-catalytic carboxyl-terminus [53]. PBD plays a pivotal role in the function of PLKs. PBD of PLK1 acts as a mediator that arranges for the kinase domain of PLK1 to be in close proximity to its substrates mainly through recognizing phosphopeptides with the core consensus motif Ser-pThr/pSer-Pro/X [54,55,56]. This phospho-epitope binding module and the PBD-dependent interaction are crucial for the proper subcellular localization of PLK1 [57]. However, PLK1 localization is not only controlled not only by PBD. The importance of the kinase domain in PLK1 targeting the centrosome and substrate recognition has been reported [58]. Although the kinase domain is not responsible for the correct localization of PLK1, it probably participates in PLK1 localization to the kinetochores [59]. It has also been suggested that the intrinsic kinase activity of PLK1 triggers its release from early mitotic structures and its relocalization to late mitotic structures [60].

In G2 cells, PLK1 is mainly localized to the nucleus and at the centrosome, and PLK1 is diffusely distributed in the cytoplasm [34,61]. In the early prophase, PLK1 remains concentrated at centrosomes and relocates to centromeres and kinetochores in the late prophase [62]. 

At the start of mitosis, the phosphorylation of cohesin subunit SA2 by PLK1 is required for the removal of cohesin complexes from chromosomal arms during prophase. It has been proposed that sororin, a cohesin-interacting protein essential for sister chromatid cohesion, is phosphorylated by CDK1 during prophase and acts as a docking protein to bring PLK1 into close proximity to SA2. This results in the phosphorylation of SA2 and subsequent removal of cohesin complexes from chromosomal arms, an event necessary for chromosome compaction [29,63]. The activation of PLK1 occurs initially in the nucleus of early G2 cells and later at centromeres during early mitosis [61,64,65]. Prior to NEBD, PLK1 localized to the centromere regions can support kinetochore maturation, and after NEBD, PLK1 enables the successful attachment of kinetochores to microtubules [66,67]. In *Xenopus* oocytes, resolving the bulk cohesion from the chromosome arms in prophase and prometaphase requires the activity of PLX1, the *Xenopus* analog of PLK1 [68].

PLK1 undergoes substantial redistribution during the transition from metaphase to anaphase, where PLK1 is subsequently relocalized from kinetochores to the central spindle [52,69]. After the onset of anaphase, PLK1 relocates to the central spindle and remains enriched at the midbody ring during the late stages of cytokinesis [68]. More specifically, PLK1 is associated with the spindle poles until metaphase, but as cells pass through the anaphase, PLK1 is relocalized to the equatorial plate, where spindle microtubules overlap in the midzone [52]. It is evident that the association of PLK1 with the spindle is highly dynamic and that PLK1 can function at multiple stages of mitotic progression.

In meiosis, similar dynamic patterns of PLK1 distribution have been observed as in mitosis. Initially, PLK1 is uniformly distributed in the cytoplasm in mouse oocytes at the germinal vesicle stage (GV). It later accumulates at microtubule organization centers (MTOCs) and kinetochores from NEBD to MI, whereas PLK1 is localized to the spindle midzone during anaphase [18]. In porcine oocytes, PLK1 is accumulated in the region of the spindle poles during the MI/MII stage, and in telophase I (TI) PLK1 localizes to the region of the spindle midzone [4]. Similarly, PLK1 was distributed over the spindle midbody in mouse oocytes during the anaphase/TI (ATI) [70], and also detected at the spindle poles during M-phase spindle assembly in rat oocytes [71]. It has been suggested that the varying subcellular localization of PLK1 is associated with its specific functions at different division stages, indicating that PLK1 may be associated with spindle organization in stages MI or MII [4]. Importantly, oocyte culture media enriched in follicle-stimulation hormone (FSH) affects global translation and spindle morphology [72], which may also be related to PLK1 expression or activity in the oocyte. PLK1 expression during oocyte maturation may determine developmental potential after fertilization. PLK1 and dynactin subunit 3 (DCTN3) were found to be expressed at lower levels in oocytes matured in vitro, which may contribute to an increased proportion of embryos with abnormal PLK1 and DCTN3 expression levels leading to reduced developmental competence [73]. With this in mind, a comparison of PLK1 and DCTN3 gene expression between oocytes of young and aged women revealed that PLK1 and DCTN3 increase with age, suggesting that defects in the expression of *Plk1* and *Dctn3* transcripts in the aged oocyte could affect the developmental potential of the embryo by increasing the risk of aneuploidy [74]. Translational profiling in oocytes from aged mice revealed a range of differentiated transcripts compared to oocytes from young females. Differentially translated mRNAs in oocytes from aged females are associated with cell cycle regulation. Specific maternal transcripts with differential translation depending on maternal age encode factors associated with meiotic spindle assembly [75].

## 5. PLK1 Regulates Cell Cycle Progression

### 5.1. PLK1 Supports Nuclear Envelope Breakdown

Nuclear pore complex (NPC) disassembly is a crucial event of NEBD and is required for nuclear envelope permeabilization [76]. In mitotic cells, the phosphorylation of NPC components by PLK1 leads to NPC disassembly and disruption of this process by PLK1 inhibition results in abnormal NEBD [17]. PLK1 has been shown to be recruited to the NPC in human cells prior to NEBD, and PLK1 localization to the nuclear envelope is required for efficient NEBD [67]. Moreover, PLK1 phosphorylates components of condensin in cultured human cancer cells, facilitating chromosome condensation and segregation [26,31]. 

It has been shown that temporal recruitment of PLK1 to chromosome arms by the ATPase PICH (PLK1-interacting checkpoint “helicase”) during prophase plays an important role in the regulation of mitotic chromosome architecture as PLK1 on the chromosome arms phosphorylates substrates, including cohesin. This results in the release of some arm cohesion and facilitates chromosome compaction by PICH in an ATPase-dependent manner [77].

In oocytes, several protein kinases, including cyclin-dependent kinase 1 (CDK1), protein kinase B (PKB), mitogen-activated protein kinase (MAPK), Aurora A kinase (AURKA), and PLK1 are involved in essential meiotic events such as the regulation of NEBD and cell cycle progression [18,46,78,79,80,81,82]. Similarly to mitosis, in mouse oocytes, PLK1 has been shown to promote both NEBD and chromosome compaction independently of CDK1 [18,24]. It has been revealed that in Xenopus oocytes, PLX1 phosphorylates and inhibits the activity of Myt1, the CDK1 inhibitory kinase [83]. In mouse oocytes, PLK1 is localized uniformly in the cytoplasm at the GV stage, at kinetochores and MTOCs from NEBD to metaphase I, and in anaphase I, it is detected at the spindle midzone [14,70,84]. In mouse spermatocytes, PLK1 was detected at the centrosomes of meiotic spindles, indicating a potential role of PLK1 in the regulation of the meiotic spindle dynamics, as it occurs in mitosis [85]. In mouse oocytes, spindle assembly occurs through the self-assembly of more than 80 MTOCs formed during prophase from a cytoplasmic microtubule network that functionally replaces centrosomes [86]. PLK1 is activated at MTOCs, and its activity is required for efficient meiotic resumption by promoting NEBD [18] (Table 1). Notably, PLK1 triggers decondensation of the MTOCs’ structure, and MTOCs fragment after NEBD fuse on either side of the condensed bivalents to form bipolar spindles [18,20]. Interestingly, NEBD occurs earlier in oocytes from females of advanced age than in their younger counterparts [87], which may be related to a differential expression or activity of PLK1 in oocytes from different maternal age groups.

### 5.2. PLK1 Activates Anaphase-Promoting Complex/Cyclosome (APC/C)

Cell cycle progression requires the activity of the cyclin B/CDK1 complex and its downstream targets, including the anaphase-promoting complex/cyclosome (APC/C), the ubiquitin ligase that mediates the degradation of cyclin A, cyclin B, and securin [88,89]. Several PLK1 phosphorylation sites have been identified on APC/C, and PLK1 facilitates the activation of APC/C in the presence of active cyclin B/CDK1 in vitro [32,90]. Nevertheless, the detailed mechanism for PLK1 regulation of APC/C activation remains unclear. However, it has been shown that the effect of PLK1 on APC/C activity is essential in both mitosis and meiosis [18,91] (Table 1). PLK1 colocalizes with early mitotic inhibitor 1 (EMI1) at mitotic spindle poles and is essential for EMI1 destruction [33]. EMI1 is required for cyclin B accumulation and regulates mitosis by inhibiting the premature activation of APC/C, so the destruction of EMI1 is required for mitotic exit [92]. Thus PLK1 is thought to indirectly activate APC/C by promoting the destruction of EMI1, an event that leads to mitotic exit [93]. In mouse oocytes, PLK1 also promotes EMI1 degradation, suggesting that PLK1 regulates the activation of APC/C in meiosis [18] (Figure 2). Similarly, PLK1 phosphorylates EMI2, another APC/C inhibitor; the phosphorylation of EMI2 leads to its degradation [35]. EMI2 is one of the cytostatic factors which represses APC/C activity by binding to APC/C-CDC20. Unlike EMI1, which acts and inhibits APC/C in the interphase, EMI2 is expressed at the beginning of the MII stage and rapidly decreases after fertilization (Figure 2) [94,95,96]. Similar data were also presented for *Xenopus* oocytes [97,98].

### 5.3. The Role of PLK1 in Chromosome Segregation and Spindle Formation

The activity of PLK1 is important for centrosome formation in mitotic cells. It has been proposed that centrioles generate a local pulse of PLK1 activity prior to entry into mitosis that initiates centrosome maturation [99]. PLK1 also activates a number of kinases that regulate centrosome maturation processes (reviewed in [100]) (Table 1). PLK1 phosphorylates several centrosomal proteins, such as ninein-like protein, CEP55, NEDD1, and pericentrin [21,22,23,101]. In addition, PLK1 contributes to centrosome maturation by expanding the matrix of pericentriolar material (PCM) and docking the γ-tubulin complex, two PLK1-dependent processes that work together to increase the nucleation capacity of centrosomes for spindle assembly [102]. PLK1 is essential for centrosome separation because PLK1 phosphorylates numerous key proteins involved in this process, including the kinase Nek9 and the mitotic kinesin Eg5 [103].

The proper segregation of mitotic chromosomes requires proper bipolar attachment of sister chromatids to microtubules originating from opposite spindle poles. The phosphatase-associating protein Apolo1 has been shown to interact with PLK1 and control PLK1 kinase activity to ensure accurate chromosome segregation [104]. It is known that somatic cells cannot achieve correct chromosome alignment without PLK1 activity [13]. In human cells, the inhibition of PLK1 activity resulted in spindle-dependent rupture of the centromere, suggesting that centromere DNA threads induced by PLK1 inhibition are likely caused by abnormal elongation of the centromere’s nuclear chromatin by spindle traction forces [105]. PLK1 has been suggested to play a role in maintaining the integrity of the centromere for chromosome alignment in addition to its existing role in spindle stabilization [105].

PLK1 promotes meiotic progression in oocytes and is required for high-quality meiotic spindle formation [14,18]. Moreover, PLK1 activity is essential for spindle integrity and proper chromosome segregation during meiosis I [28]. In mouse oocytes, PLK1 accumulation has been detected at kinetochores, along bivalent chromosomes, and at MTOCs [18,20,24] (Table 1; Figure 2). Although Plk1 conditional knockout (cKO) mouse oocytes are capable of undergoing NEBD, they exhibit abnormal chromosome compaction, do not form normal bipolar MI spindles, and exhibit defects at MTOCs [24]. Notably, PLK1 triggers the decondensation of MTOCs by releasing the MTOC linker protein C-NAP1 [20,24] (Table 1). The inhibition of PLK1 kinase or RNAi-mediated PLK1 depletion resulted in a suppressed dissociation of C-NAP1 from MTOCs, induced a delay in chromosome compaction, and caused defects in the fragmentation of MTOCs in oocytes [20]. This event is mediated by the activity of AURKA, which is required for full PLK1 activation and subsequent induction of the release of C-NAP1 from MTOCs in mouse oocytes [46]. Subsequently, MTOCs fragmented after NEBD fuse on either side of the condensed bivalents to form bipolar spindles [18,20]. Furthermore, PLK1 is required for the normal localization of acentriolar MTOC components such as gamma-tubulin, CEP192, and NEDD1 in oocytes [24] (Table 1). These data indicate that the fragmentation of MTOCs in mammalian oocytes after NEBD is regulated by PLK1 activity. In contrast to the role of PLK1 in acentriolar oocytes, and apart from its role in the formation of meiotic bipolar spindles in spermatogenesis, PLK1 is involved in the assembly of the pericentriolar matrix and in the maturation and migration of meiotic centrosomes [85]. 

Another notable role of PLK1 has been reported in the context of the recently discovered fluid-like meiotic spindle domain (LISD), a subcellular structure in mammalian oocytes that penetrates the spindle poles and concentrates multiple regulatory microtubule factors so that they can rapidly diffuse into the spindle volume [24,106]. In PLK1 cKO oocytes, an irregular distribution of LISD factors was found, suggesting that PLK1 is essential for LISD assembly [24]. One of the possible components of LISD in mammalian oocytes is ankyrin 2 (ANK2), which has been recently found to be localized to the spindle region of mouse oocytes [107]. In summary, the roles of PLK1 in mitosis and meiosis are comparable. PLK1 influences almost every step of cell division: entry into the M phase, chromosome condensation, NEBD, spindle organization, chromosome segregation, and cytokinesis. In addition, PLK1 is also involved in the regulation of the translational machinery.

## 6. The Role of PLK1 in mRNA Translation

### 6.1. PLK1 Acts in Translational Machinery

**Figure 3:**
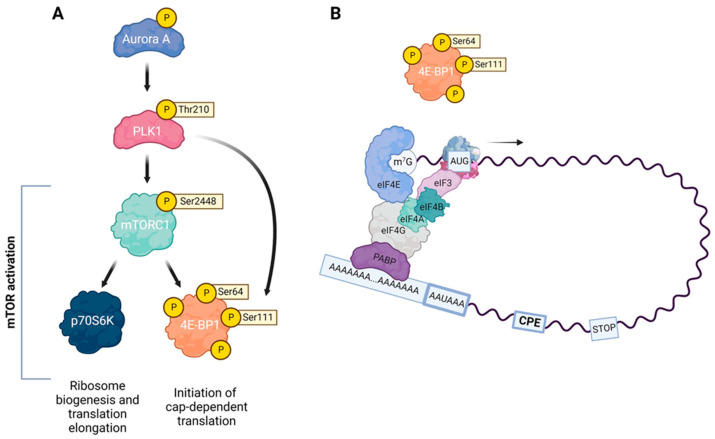
Role of PLK1 in the initiation of translation. (**A**) Activation of polo-like kinase 1 (PLK1) requires phosphorylation of conserved threonine residue (Thr 210) by aurora kinase A (AURKA). Later PLK1 phosphorylates the mammalian target of rapamycin complex 1 (mTORC1), which plays a major role in the phosphorylation and inactivation of eukaryotic translation initiation factor 4E (eIF4E)-binding protein 1 (4E-BP1) and in the initiation of cap-dependent translation. Moreover, PLK1 also regulates the phosphorylation of the 4E-BP1 at the Ser111 residue located on the spindle, enabling the local translation of spindle-associated proteins. Phosphorylated mTORC1 activates p70S6 kinase (p70S6K), resulting in ribosome biogenesis and translation elongation. (**B**) Scheme of cap-dependent translational initiation after poly(A) elongation. MTORC1 hyperphosphorylates 4E-BP1, which results in the dissociation of 4E-BP1 from the cap-binding subunit eIF4E. Released eIF4E together with the RNA helicase eIF4A and large adaptor protein eIF4G form the eIF4F complex, which binds to the m7G cap of mRNA. Poly(A)-binding protein (PABP) binds to the elongated poly(A) tail and eIF4G, forming an mRNA loop and stabilizing the cap. EIF4B interacts with and promotes eIF4A activity and interacts with the eIF3A subunit of the 43S preinitiation complex. After 5′ UTR scanning and AUG recognition, the complex recruits a 60S ribosomal subunit and starts protein synthesis.

Regulation of mRNA translation plays an important role in the control of gene expression. More than 30 years ago, it was shown that protein synthesis in mouse oocytes is not required for NEBD during the resumption of meiosis but is essential for the proper formation of the meiotic spindle and progression to MII [108]. Therefore, mammalian oocytes represent a good model to study translational control in the context of meiotic cell cycle progression and, in particular, spindle formation. PLK1 has been shown to be an active player in translational control in mammalian oocytes [27] (Figure 3). In many cell types, including oocytes, localized translation ensures that mRNA is translated in place, providing an additional mechanism of protein regulation within a cell [109,110]. It has been suggested that translation in somatic cells occurs preferentially in subcellular compartments, creating translation hotspots for specific mRNAs [111,112]. In mouse oocytes, RNA-rich domains and translation hotspots develop during the first meiosis in the chromosomal region and in the region previously surrounded by the nucleus [113]. These translation hotspots are controlled by the activity of the mTOR (mammalian target of rapamycin) pathway. This mechanism is thought to control the temporal and spatial translation of a specific set of transcripts required for normal spindle assembly, chromosome alignment, and segregation [113].

MTOR, a serine/threonine kinase, acts in two different multiprotein complexes, mTOR complex 1 (mTORC1) and mTOR complex 2 (mTORC2) [114,115]. The regulatory associated protein of MTORC1 (RAPTOR) functions as a scaffold for mTOR kinase to recruit and phosphorylate specific substrates such as ribosomal protein S6 kinase (S6K) and eukaryotic translation initiation factor 4E binding protein 1 (4E-BP1). PLK1 is a potential regulator of mTORC1 since the inhibition of PLK1 decreased mTORC1 phosphorylation at the Ser2448 residue [116]. In addition, the knockdown of PLK1 decreased the phosphorylation of 4E-BP1 at the Thr70 residue as well as the phosphorylation of both p70S6 kinase (p70S6K) and ribosomal protein S6 (RPS6), a substrate of p70S6K [116,117]. Apparently, PLK1 controls the initiation and elongation steps of mRNA translation by regulating the phosphorylation of 4E-BP1 and p70S6K, the major targets of mTORC1 (Figure 3A). The cited data indicate that PLK1 is involved in the activation of the mTORC1 signaling pathway and thus promotes mRNA translation. However, PLK1 has been described to inhibit mTORC1 in the interphase cells under nutrient starvation and amino acid deprivation. This suggests that the functions of PLK1 in mitotic and interphase cells are mediated by different mechanisms since PLK1 inhibition increases mTORC1 activity in interphase cells but not in mitotic cells [118]. The critical checkpoint of mRNA translation is the formation of the eukaryotic translation initiation factor 4F (eIF4F) complex, which is involved in loading mRNA with ribosomal subunits and initiating mRNA translation. The eIF4F complex consists of three subunits, the scaffold protein eIF4G, the ATP-dependent helicase eIF4A, and the cap-binding protein eIF4E. 4E-BP1 negatively regulates the interaction of eIF4E and eIF4G and acts as an inhibitor of translation initiation [119] (Figure 3B). The phosphorylation of 4E-BP1 at residues Thr37/46 by the mTOR pathway promotes the phosphorylation of the other 4E-BP1 sites Thr70 and Ser65, leading to a release of 4E-BP1 from the cap-binding protein eIF4E and hence enabling the formation of the translationally active eIF4F complex [120,121].

### 6.2. PLK1 Regulates mRNA Translation in Mitosis and Meiosis

During mitosis, repression of 4E-BP1 results in misaligned chromosomes, abnormal centrosomes, and polyploidy [25]. During cell cycle progression in mitotic cells, interaction with PLK1 could directly affect the function of 4E-BP1 [25]. The binding of 4E-BP1 to eIF4E is regulated by the phosphorylation of multiple Ser/Thr residues of 4E-BP1 by upstream kinases, concomitant with cell cycle progression, hormone stimulation, and nutrient availability. For example, CDK1 phosphorylates 4E-BP1 at up to four sites during mitosis [122,123]. In human mitotic cells, PLK1 colocalizes at the mitotic spindle with the mTOR target 4E-BP1 phosphorylated at the Thr37/46 sites and phosphorylates 4E-BP1 directly at residue Ser111; the colocalization of PLK1 and phospho-4E-BP1 is essential for the regulation of spindle integrity [25]. In addition to localizing to mitotic spindles during metaphase, PLK1 colocalizes to centrosomes with 4E-BP1 phosphorylated at Thr37/46 [25]. In oocytes, 4E-BP1 phosphorylation is essential for proper meiotic progression. The localization of phosphorylated 4E-BP1 isoforms to the meiotic spindle suggests that the translation of mRNAs occurs at the spindle [124]. mRNAs associated with metaphase spindles in oocytes and in mitotic cells are crucial for the translation of spindle components and contribute to spindle formation [109,113]. In mouse oocytes, the kinases CDK1 and mTOR are the major positive regulators of the 4E-BP1 phosphorylation after NEBD [123]. It has been shown in mouse oocytes that CDK1 exerts its effect on 4E-BP1 phosphorylation via the activation of mTOR, although it cannot be excluded that CDK1 phosphorylates 4E-BP1 directly [79,124]. The inhibition of 4E-BP1 phosphorylation decreases the amount of the structural protein β-tubulin at the MI spindle, demonstrating the role of 4E-BP1 phosphorylation in spindle formation in the oocyte [27].

Similarly to mitosis, during the meiotic maturation of mouse oocytes, PLK1 phosphorylates the Ser111 residue of 4E-BP1, directly and indirectly, phosphorylates the Ser64 residue of 4E-BP1 localized to meiotic spindles; the indirect phosphorylation of the Ser64 residue is likely mediated by interactions of PLK1 with CDK1 [27]. The inhibition of PLK1 results in a loss of phospho-4E-BP1 at oocyte spindle poles and disrupts spindle functions, suggesting that PLK1 promotes the phosphorylation of 4E-BP1 at the spindle and supports spindle formation by activating mRNA translation [27]. These data suggest that PLK1 regulates 4E-BP1 activity in both mitosis and meiosis, mediating the translation of spindle-associated proteins that control spindle assembly and stability. On the other hand, PLK1 indirectly activates p70S6K, which promotes ribosome biogenesis and translation elongation by phosphorylating ribosomal protein S6 (RPS6) [125] (Figure 3A). p70S6K, a marker of active mRNA translation, has been detected around condensing chromosomes [109]. In addition, the translational repressor 4E-BP1 was colocalized with the p70S6K substrate RPS6 to metaphase spindles in mouse oocytes [109]. PLK1 also regulates the phosphorylation of 4E-BP1 at the Ser111 residue located on the spindle, enabling the local translation of spindle-associated proteins [126].

These data suggest that PLK1 affects the initiation and elongation steps of mRNA translation by phosphorylating the regulator of mRNA translation 4E-BP1 and regulating the activity of mitogen-activated p70S6K. As a key regulator of oocyte meiosis and mRNA translation, PLK1 is an important determinant of oocyte quality.

## 7. Conclusions

PLK1 is a significant player in cell division, regulating a number of crucial steps in mitosis and meiosis. The dynamic control of PLK1 activity is essential for its role in centrosome maturation, spindle assembly, microtubule and kinetochore binding, and cytokinesis. During mitosis and meiosis, PLK1 regulates the localized translation of spindle-enriched mRNAs by phosphorylating a downstream target of the mTOR pathway, the translational repressor 4E-BP1. It is evident that PLK1 plays an important role in the localized translation of specific transcripts required for proper spindle assembly and function. Further efforts to identify mRNAs whose translation is controlled by PLK1 will open up new possibilities for regulating the progression of mitosis and meiosis. In particular, the analysis of PLK1 expression in aging oocytes will provide new valuable findings that will be beneficial in the field of human reproduction.

## Figures and Tables

**Table 1:** Polo-like kinase 1 (PLK1) is implicated in the key phases of mitosis and meiosis.

Cell Cycle Stage	PLK1 Role in Mitosis	Reference	PLK1 Role in Meiosis	Reference
M-phase entry	PLK1 activity is required for entry into mitosis in vertebrates	[1,2,13]	PLK1 activity is essential for progression through the M-phase	[14]
M-phase entry	PLK1-dependent phosphorylation of phosphatase CDC25C promotes mitotic entry	[7]	PLK1 is required for activation of the phosphatase CDC25C and CDK1	[15]
NEBD	PLK1 participates in nuclear envelope reformation and nuclear pore disassembly	[16,17]	PLK1 promotes NEBD during meiotic resumption	[18]
NEBD	PLK1 triggers chromosome condensation in early mitosis	[8,9]	PLK1 triggers chromosome compaction	[19]
Spindle organization	PLK1 activates Aurora B, Haspin, and BUB1 in early mitosis, which establishes the centromere and recruits the chromosomal passenger complex	[6]	PLK1 triggers decondensation of the MTOC structure and is required for C-NAP1 dissociation during NEBD, which is critical for the fragmentation of MTOC components	[20]
Spindle organization	PLK1 phosphorylates several centrosomal proteins, including ninein-like protein, CEP55, NEDD1, and pericentrin	[21,22,23]	PLK1 regulates MTOC fragmentation and LISD recruitment. PLK1 controls the normal localization of acentriolar MTOC components (γ-tubulin, CEP192, NEDD1)	[24]
Spindle organization	PLK1 colocalization with p4E-BP1 on the spindle is essential for spindle integrity	[25]	PLK1 recruits centrosomal proteins to acentriolar MTOCs to promote spindle formation	[18]
Spindle organization	PLK1 is essential in spindle assembly checkpoint and chromosome segregation	[26]	PLK1 regulates 4E-BP1 phosphorylation on the spindle, which is essential for spindle formation integrity	[27]
Segregation of chromosomes	PLK1 stabilizes kinetochore-microtubule attachment and spindle checkpoint silencing	[10,26]	PLK1 plays an essential role in chromosomal segregation and spindle formation in porcine oocytes	[4,28]
Segregation of chromosomes	PLK1 phosphorylates Sororin and SA2, a key component of the cohesion dissociation pathway	[8,29]	PLK1 plays an essential role in stable kinetochore-microtubule attachment	[18]
Cytokinesis	PLK1 is an essential early regulator of anaphase spindle elongation and cytokinesis	[2,30]	PLK1 is required for mono-orientation and the protection of centromeric cohesion	[31]
Cytokinesis	PLK1 phosphorylates and activates APC/C and controls the destruction of APC/C inhibitor EMI1	[32,33,34]	PLK1 activates APC/C by promoting degradation of the APC/C inhibitors EMI1 and EMI2, an essential event for entry into anaphase 1	[18,35]
